# Evaluation of the Antimicrobial, Thermal, Mechanical, and Barrier Properties of Corn Starch–Chitosan Biodegradable Films Reinforced with Cellulose Nanocrystals

**DOI:** 10.3390/polym14112166

**Published:** 2022-05-26

**Authors:** Claudio Alonso Díaz-Cruz, Carolina Caicedo, Enrique Javier Jiménez-Regalado, Ramón Díaz de León, Ricardo López-González, Rocio Yaneli Aguirre-Loredo

**Affiliations:** 1 Departamento de Ingeniería Química, Facultad de Ciencias Químicas, Universidad Autónoma de Coahuila, Blvd. Venustiano Carranza SN, Saltillo 25280, Coahuila, Mexico; alonsotv12@hotmail.com; 2 Grupo de Investigación en Química y Biotecnología (QUIBIO), Facultad de Ciencias Básicas, Universidad Santiago de Cali, Pampalinda, Santiago de Cali 760035, Colombia; carolina.caicedo03@usc.edu.co; 3 Departamento de Procesos de Polimerización, Centro de Investigación en Química Aplicada (CIQA), Blvd. Enrique Reyna Hermosillo 140, Saltillo 25294, Coahuila, Mexico; enrique.jimenez@ciqa.edu.mx (E.J.J.-R.); ramon.diazdeleon@ciqa.edu.mx (R.D.d.L.); ricardo.lopez@ciqa.edu.mx (R.L.-G.); 4 Investigadora por México CONACYT-Centro de Investigación en Química Aplicada Blvd. Enrique Reyna Hermosillo 140, Saltillo 25294, Coahuila, Mexico

**Keywords:** biodegradable film, corn starch, chitosan, cellulose nanocrystals, antimicrobial film, nanocomposite

## Abstract

Packaging materials play an essential role in the preservation and marketing of food and other products. To improve their conservation capacity, antimicrobial agents that inhibit bacterial growth are used. Biopolymers such as starch and chitosan are a sustainable alternative for the generation of films for packaging that can also serve as a support for preservatives and antimicrobial agents. These substances can replace packaging of synthetic origin and maintain good functional properties to ensure the quality of food products. Films based on a mixture of corn starch and chitosan were developed by the casting method and the effect of incorporating cellulose nanocrystals (CNC) at different concentrations (0 to 10% *w*/*w*) was studied. The effect of the incorporation of CNC on the rheological, mechanical, thermal and barrier properties, as well as the antimicrobial activity of nanocomposite films, was evaluated. A significant modification of the functional and antimicrobial properties of the starch–chitosan films was observed with an increase in the concentration of nanomaterials. The films with CNC in a range of 0.5 to 5% presented the best performance. In line with the physicochemical characteristics which are desired in antimicrobial materials, this study can serve as a guide for the development this type of packaging for food use.

## 1. Introduction

Plastic materials are widely used globally due to their low production cost and excellent barrier, mechanical and thermal properties. Such synthetic plastics are used in various areas and products, such as food packaging, supermarket bags, toys, electronic devices, kitchen appliances, automotive parts and medical devices, among many others. The extensive use of plastic materials derived from petroleum, coupled with their inadequate disposal, has negatively impacted the environment. It is estimated that the global consumption of plastic exceeds 700 million tons per year [1]. To reduce and avoid this environmental problem, the use of polymers obtained from renewable sources to produce plastic materials is being more widely considered, mainly due to their rapid biodegradation and viability of being compostable. Polymers that can produce biodegradable packages/films can be polysaccharides such as starch, chitosan, cellulose and their derivatives, alginate and pectin; proteins such as gelatin, zein, and collagen; or polyesters such as polyhydroxybutyrate, polylactic acid, polycaprolactone [2,3,4,5,6].

The use of starch as a packaging material has been widely investigated in recent years due to its high availability in nature, multiple sources of production, and economic viability [7,8,9]. Meanwhile, chitosan, i.e., a biopolymer obtained from waste from the fishing industry, is a product with significant added value which can be obtained in large quantities [10,11,12]. It has been reported that the protonated amino groups of chitosan promote the formation of intermolecular bonds with the structural matrix of starch films, giving rise to a material with better mechanical performance, good thermal stability, and better water resistance [13,14]. This biopolymer has demonstrated antimicrobial, antifungal, anti-inflammatory, and antioxidant properties, making it an excellent alternative for the production of active materials for various industries such as food, packaging, and medicine [12,15]. Chitosan is a widely investigated polymer for the preparation of composite materials due to its excellent compatibility with compounds of both natural and synthetic origin, promoted by amino, hydroxyl, and carboxyl groups [13,15]. With the mixture of both biopolymers, new, low-cost packaging materials could be generated that could also improve the quality of packaged products.

Recent studies have evaluated the use of nanometric compounds for the reinforcement of materials made from natural polymers. It has been found that reducing the particle size of the reinforcing materials promotes more homogeneous dispersion and increases the specific surface of the reinforcements, such as nanocellulose and nanoclays [16,17,18,19]. Nanocellulose is produced from cellulose, the most abundant polymer in nature, which can be obtained from the residues of agricultural byproducts. Cellulose is subjected to processes that can be enzymatic, mechanical, or chemical, in which its amorphous regions are eliminated, giving rise to a more crystalline and ordered nanometric structure [20,21,22]. Nanocellulose is a renewable material which is friendly to the environment, and due to its size, it can provide exciting advantages for packaging materials. The use of nanocomposites improves the thermal, mechanical, and barrier properties of packaging materials by using lower reinforcement loads (1 to 5% by volume) compared to when micrometric or larger size reinforcements are used [23]. In addition to improving the physicochemical properties of the materials, some nanocomposites have exhibited significant antimicrobial and antioxidant capacities, which would benefit the food packaging industry [5,17,22,24,25]. The food industry is currently trying to transition to cleaner products by reducing the number of additives and preservatives of synthetic origin, promoting research using various types of preservatives and antibacterials. The use of nanoparticles has been widely investigated as a substitute for various antibacterials, mainly to reduce and combat the global problem caused by the extensive use of antibiotics, which generate resistance in microorganisms [26,27] The antibacterial activities of nanomaterials depend on the properties of the nanoparticles and the bacteria of interest. Therefore, more research should be carried out, since there are many types of nanoparticles with different effects on microorganisms, depending on their nature, morphology, size, and composition.

Various studies have been carried out on the development of packaging materials using natural polymers in pure form or as mixtures, as well as nanometric materials for the improvement of physicochemical properties. The polymer–nanomaterial relationship plays a fundamental role in the development, functional properties, and application of such materials. Previously, it was possible to develop starch–chitosan composite materials with good handling and barrier characteristics; however, when seeking to incorporate a nano-sized material such as cellulose crystals, the information reported in the literature was inconsistent (0.5 to 50% *w*/*w*) [20,23,28,29]. This is due to the wide ratio when nanomaterials are incorporated into packaging materials, since their effect depends on the type of polymer, its composition, and its interactions with the matrix. As such, each nanomaterial must be evaluated to find the appropriate concentration for the material to be used.

The objective of this study was to evaluate the antimicrobial, thermal, water vapor barrier, and mechanical performance of corn starch–chitosan-based films when a reinforcing material such as cellulose nanocrystals (CNC) was incorporated at different concentrations (0, 0.5, 2.5, 5, 7.5, and 10% *w*/*w* biopolymers). Nanocrystals could substantially improve the properties of these biodegradable films and, in the future, be a viable alternative to synthetic food packaging.

## 2. Materials and Methods

### 2.1. Materials

Corn starch (Grain Processing Corporation, Muscatine, IA, USA) and chitosan (90% deacetylation degree, Alfadelta Materias Primas, Naucalpan, Mexico) were used without modification. Glycerol (J.T. Baker, Ciudad de Mexico, Mexico) as a plasticizing agent. Glacial acetic acid was acquired at Productos Quimicos Monterrey (Monterrey, Mexico). Cellulose nanocrystal (CNC) powder was provided by CelluForce Inc. (spray-dried powder, needle-shaped, nano-sized 7.5 nm × 150 nm, named CelluForce NCC^®^. Montreal, QC, Canada). To simulate environments with different relative humidities (11, 22, 32, 43, 57, 75, 84, and 90%), supersaturated salt solutions of LiCl, CH_3_COOK, MgCl_2_, K_2_CO_3_, NaBr, NaCl, KCl, and BaCl_2_ were used, which were purchased from Jalmek Cientifica (Monterrey, Mexico). Trypticase soy agar, casein peptone, and trypticase soy broth manufactured by MCD LAB S.A. of C.V. (Oaxaca, Mexico) were used for the cultivation of microorganisms in antimicrobial tests.

### 2.2. Film Forming Solutions Preparation

For the preparation of the polymeric matrix, solutions of acetylated corn starch (5% *w*/*v* dispersed in water) and chitosan at 1% (*w*/*v*) dissolved in acetic acid (1% *v*/*v*) were used. A 50:50 ratio of polymeric solutions and 30% glycerol was used; these concentrations were determined according to the results obtained in a previous study [13,26]. First, the starch dispersion was prepared, and the plasticizer was added. Then, the temperature was increased. When the temperature reached 50 °C, the chitosan solution (prepared by dissolving the polymer at room temperature and stirring for 24 h) was added. Cellulose nanocrystals (CNC) were added at concentrations of 0, 0.5, 2.5, 5, 7.5, and 10% (*w*/*w*) with regard to the total biopolymer content. The composite film-forming solutions were constantly stirred and maintained at a temperature of 90 °C for 10 min to achieved adequate gelatinization of the corn starch. They were then left to stand for 30 min to eliminate air bubbles that had formed during the process. The polymer nanocomposite solutions (0.75 mL/cm^2^) were poured into acrylic molds and dried in an oven (Thermolyne, Blue M, Blue Island, IL, USA) at 65 °C for 5 h. Composite films with 0% CNC were used as the control.

### 2.3. Rheology

The apparent viscosity of the filmogenic solutions was determined as a function of the nanocrystal content. A controlled stress rheometer (MCR 501 model, Anton Paar Physics, Graz, Austria) with a stainless steel cone-plate geometry of 50 mm diameter and angle of 2° was used, at a shear rate from 0.01 to 1000 s^−1^ [27]. The analysis was performed at 25 °C.

### 2.4. Antimicrobial Activity

Pathogenic bacteria *Staphylococcus aureus* and *Listeria monocytogenes*, which are of significant concern in the food industry, were used to determine the antimicrobial capacity of the composite films with various contents of CNC. The bacterial strains were inoculated following the methodology proposed by Gómez-Aldapa, Díaz-Cruz [28]. For the evaluation of the films, the disc diffusion technique was used, using film samples of 5 mm in diameter placed directly on the inoculated trypticase soy agar plates. The plates were incubated at 35.5 °C for 24 h. The evaluations were carried out in triplicate, and the results are expressed as mean and standard deviation.

### 2.5. Morphology by SEM

The morphology of the top-surface of the films was observed to determine if there was any change in the texture with an increase in the content of nanocellulose. This was performed with a SM-510 scanning electron microscope (TOPCON, Tokyo, Japan) at 5 kV at a working distance of 18 mm. Composite films were gold-palladium coated for 90 s on a Denton Vacuum model DESK II sputter (Moorestown, NJ, USA).

### 2.6. Thermal Properties

The effect of incorporating the CNC on the thermal resistance of the starch–chitosan films was determined using a DSC 2500 Discovery differential scanning calorimeter (TA Instruments, New Castle, DE, USA) in a temperature range of 0 to 250 °C, in a nitrogen atmosphere (50 cm^3^ min^−1^).

### 2.7. X-ray Diffraction

To determine if the presence and increase of the nanomaterial content modified the structural arrangement of the polymeric matrix, the latter was determined using a Siemens D500 powder diffractometer (Munich, Germany), according to the methodology suggested by Fonseca-García, Jiménez-Regalado [29].

### 2.8. Water Vapor Adsorption Isotherms

The water vapor absorption capacity of the materials was evaluated at room temperature (≈25 °C) according to the methodology proposed by Jiménez-Regalado, Caicedo [13], in a relative humidity (RH) range of 11 to 90% (*a_w_* = 0.1 to 0.9). The saline solutions were prepared according to the methodology of Aguirre-Loredo, Rodriguez-Hernandez [30]. The experimental data were fitted to the Guggenheim–Anderson–de Boer (GAB) mathematical model (Equation (1)). In Equation (1), *X* is the moisture content absorbed by the material, *Xm* is the content of water that adheres directly to the material, forming the first layer of molecules, also called monolayer; *C* is a constant related to the sorption in the monolayer; and *K* is a constant associated with the sorption of water molecules in the layers after the monolayer, which forms multilayers [30].

(1) $$X=\frac{X_{m}CKa_{w}}{\left(1-Ka_{w}\right)\left(1-Ka_{w}+CKa_{w}\right)}$$

### 2.9. Mechanical Properties

The mechanical properties of tensile strength and elongation at break of the materials were determined in a texture analyzer (TA.XT Express Enhanced, Stable Micro Systems, Godalming, UK) equipped with clamps. The films were cut into strips 1 cm wide by 6 cm long, with a gap of 3 cm between clamps. The equipment operated at a speed of 1 mm·s^−1^ [13,31]. Results are reported as the mean and standard deviation of at least ten replicates.

### 2.10. Water Vapor Permeability

Water vapor permeability (WVP) was evaluated according to the methodology proposed by Jiménez-Regalado, Caicedo [13] and following the desiccant method [32]. A glass permeability test dish containing silica gel was used, on which the biodegradable films were mounted. The test dish was placed in a desiccator with NaBr, which generated a pressure gradient of 2854.23 Pa. The test dishes were weighed every 40 min for 8 h. WVP are reported as the mean and standard deviation of three replicates.

### 2.11. Statistical Analysis

Experimental data were analyzed for statistical significance by analysis of variance (ANOVA) and Tukey’s test with a *p* < 0.05 significance level with the help of statistical software OriginPro 8.5.0 SR1 (OriginLab Corporation, Northampton, MA, USA).

## 3. Results and Discussion

### 3.1. Rheology Behavior of Film Forming Solutions

**Figure 1:**
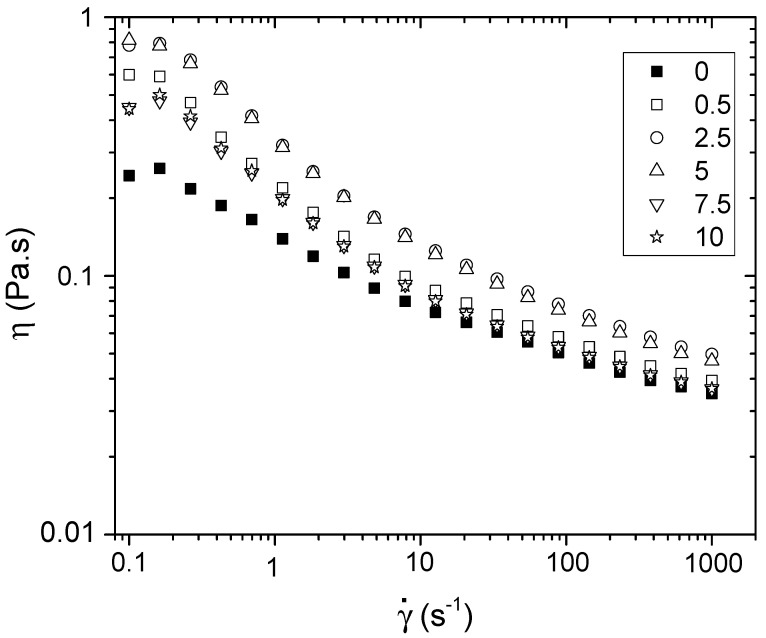
Log-log viscosity versus shear rate of film forming solutions of corn-starch and several ratios of CNC (*w*/*w*).

A rheological study of the biopolymeric film forming solutions was carried out to examine the behavior of the nanocrystals and their effect on viscosity. Figure 1 shows the apparent viscosity behavior as a function of the shear rate of the filmogenic solutions. The results showed that the apparent viscosity of the filmogenic solutions of starch–chitosan mixtures strongly depended on the shear rate they were subjected to. The decrease was due to the disentangling of polymeric chains. Similar behavior was observed in other formulations based on chitosan, both alone and in mix with other biopolymers [33]. The addition of the cellulose nanocrystals increased the apparent viscosity of the filmogenic solutions when these did not exceed a concentration of 5%. This was probably due to crosslinks generated by the CNC forming networks that caused resistance to flow. At higher percentages of nanomaterial, the viscosity no longer increased; it remained in a range similar to that of low concentrations of CNC, but always higher than that of the control film.

### 3.2. Antimicrobial Activity of the Nanocomposite Films

A package or film that is going to be in contact with food, in addition to protecting it from mechanical damage, can also prevent or delay the growth of spoilage microorganisms and some pathogenic microorganisms. In this study, the antimicrobial capacity of CNCs was evaluated when they were incorporated into corn starch–chitosan composite films. Two pathogenic microorganisms, *Listeria monocytogenes* and *Staphylococcus aureus*, were used due to their relevance to the food industry. The size of the inhibition halos (clear areas around the disks) generated by the nanocomposite film samples are presented in Table 1. An evident inhibition of the growth of both microorganisms was observed when the percentage of nanocomposites was low (0 to 5% *w*/*w*). The inhibitory effect of chitosan in the corn starch–chitosan composite film was confirmed in both *L. monocytogenes* and *S. aureus*; this test was consistent with results presented in other reports [34,35]. Chitosan is a biopolymer that has been shown to have some antibacterial capacity against both gram-positive and gram-negative bacteria [36,37]. This good antimicrobial effect has improved biodegradable materials made from natural polymers such as starch from several sources [12,38].

The maximum inhibitory effect was observed on *L. monocytogenes*, which showed the most extensive diameter inhibition halo (12.52 ± 0.01 mm.) when a concentration of 0.5% CNC was used. In this case, the antimicrobial effect of the content of CNC was demonstrated in contrast to the control. In *S. aureus*, on the other hand, although the evaluations showed an inhibitory effect of the starch–chitosan films with CNC concentrations lower than 5.0%, the impact was not more significant than that shown in the control films, revealing a difference in the sensitivity of both gram-positive microorganisms.

The nanomaterials (NM) or nano reinforcement composition, shape, and size play an essential role in their antibacterial capacity. The smaller the NMs, the greater their specific surface area, with the latter increasing the probability of interacting with and crossing the bacterial cell membrane [23,26,39,40].

The mechanism by which nanoparticles generate toxicity is unknown; however, efforts are still being made to elucidate it. One possible mechanism is adhesion by the electrostatic interaction of nanoparticles to the bacterial cell membrane, affecting its structural integrity [25,38]. Some NMs can also cause oxidative stress through the formation of free radicals, which alters the permeability of the cell membrane, damaging it and causing its death [24,25]. The use of nanometric-sized compounds incorporated in packaging materials has been investigated in recent years due to their interesting functional properties.

The use of nanomaterials with antimicrobial effects offers several advantages and has broad applications. Benefits include prolonged antimicrobial activity, very low environmental or health toxicity and the absence of resistance of microbes to antibiotics and other drugs [38]. These antimicrobial biopolymeric formulations can be used to develop materials for the food packaging industry, as well as in the development of various medical devices.

### 3.3. Surface Morphology

**Figure 2:**
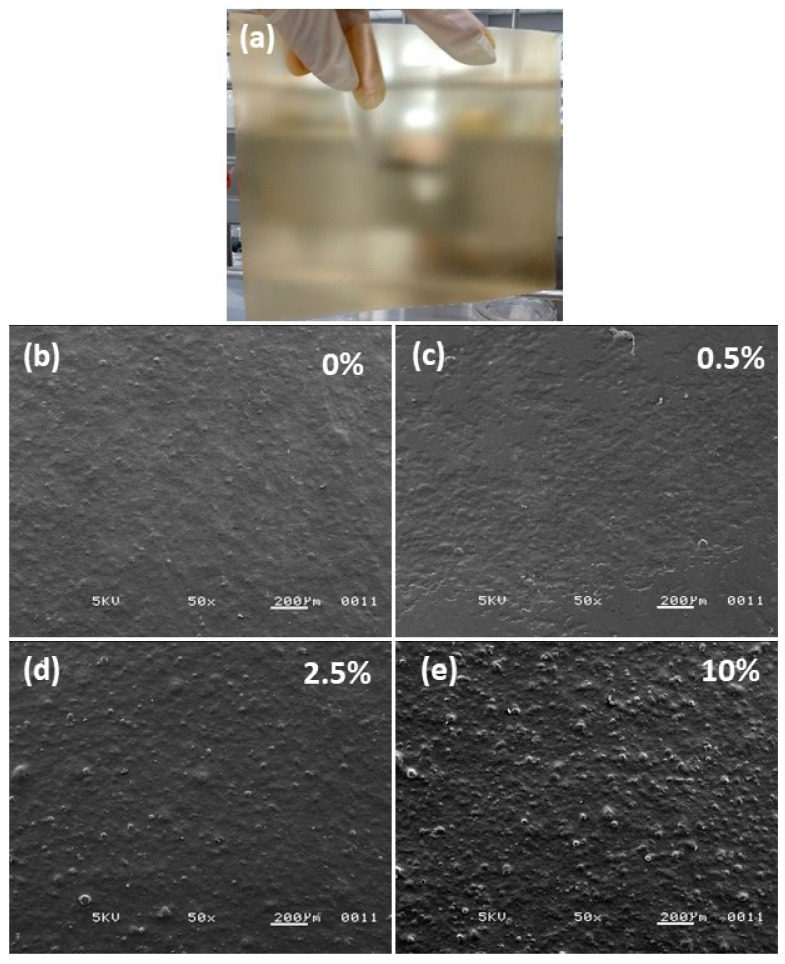
(**a**) Photograph and top surface morphology of corn starch–chitosan films with cellulose nanocrystals (CNC) at (**b**) 0%, (**c**) 0.5%, (**d**) 2.5%, and (**e**) 10% (*w*/*w*).

Visually, there was no change in the appearance of the starch–chitosan films with incorporated nanomaterials, nor with the increase in their concentration (Figure 2a, only one photography of the biodegradable materials is presented because those obtained with the other formulations were visually the same). However, when observed with a scanning electron microscope (SEM), a change in their morphology was observed with the addition of CNC. The top surface morphology of the films is shown in Figure 2.

The control film (Figure 2b), that is, the film containing no nanomaterials, presented a more homogeneous appearance. An increase in surface roughness or heterogeneity was observed in the presence of CNC. The surface appeared to be better structured and less rough with low concentrations of CNC (0.5 and 2.5% *w*/*w*), as seen in Figure 2c,d. In contrast, with higher CNC content, the films had a more irregular surface (Figure 2e), which may be a consequence of the reduction in the dispersibility and an aggregation of nanomaterials [6,19,20,41]. This change in the morphology of the composite films with the addition of CNC was similar to that reported by Chen, Shi [20] for cassava starch films with cellulose crystals in micro- and nano-sizes.

### 3.4. Thermal Properties

**Figure 3:**
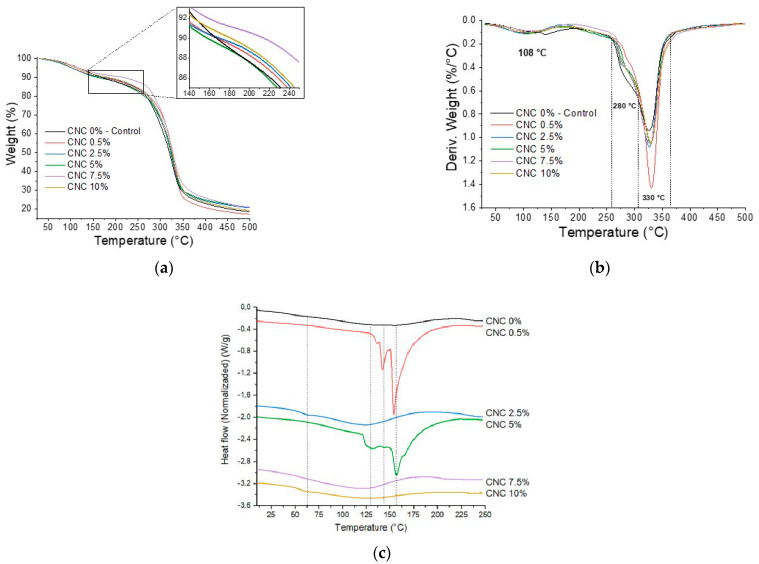
(**a**) TGA, (**b**) DTG curves, (**c**) DSC thermogram of biopolymer (corn starch–chitosan) and nanocomposite films incorporated with different concentrations of CNC (0 to 10% *w*/*w*).

Figure 3a,b show thermogravimetric analysis curves and their respective derivatives that detail the behavior of biopolymeric samples with varying contents, i.e., between 0 and 10%, of CNC. In general, the biopolymers had similar degradation temperatures (~300 °C); however, the plasticizer content and the addition of particles have been shown to significantly influence the thermal behavior at processing temperatures between 120 °C and 200 °C.

In this study, the plasticizer content was constant. As such, the addition of nanoparticles maintained stability and, in some cases, improved it. The first zone of mass loss corresponded to the evaporation of water. Later, in the extended zone between 150 °C and 250 °C, a more pronounced negative slope (abrupt fall) of the control sample was observed. This second area represents the outlet of the plasticizer. This behavior could be explained by the interaction between the CNC and the plasticizer [13,20], or by the barrier effect of CNC that caused a delay in the release of the plasticizer [16]. Likewise, the degradation temperatures in the range of 255 °C and 365 °C (Figure 3b) presented bifurcation due to the degradation of each polymer. Similar degradation processes and degradation temperature ranges have been reported for other biomaterials based on starch and chitosan with cellulose nanoparticles [20,39]. The effect on the CNC sample was 0.5% (containing a low percentage of CNC). This result is consistent with other studies that described an adequate interaction through the formation of hydrogen bonds between the hydroxyl groups of cellulose and starch [40] and between the hydroxyl groups and the amino groups of chitosan [34].

The DSC thermogram (Figure 3c) shows curves with an amplified band between ~50 °C and ~160 °C, characteristic of partially gelatinized and plasticized starch materials. The transitions found in pure polymers are reported in a previous work showing Tg for starch and chitosan of around 57 °C and 112 °C, respectively [13]. In the same way, a chitosan-starch blend in the development of films by casting, which implies the prior mixing of film-forming solutions, revealed optimal interaction, resulting in a homogeneous morphology. Thus, a calorimetric analysis showed a decrease in the enthalpy of gelatinization (Table 2) when the destructuring of the starch granules was promoted with acid solutions (acetic acid was used in the preparation of the chitosan film-forming solution) [41]. The values of the glass transition temperature (T_g_) are of low intensity (Table 2); however, the samples increased with the increase in the concentration of CNC due to the restriction in the mobility in the amorphous regions of the starch that generated intermolecular interactions between the nanocrystals and the matrix. On the other hand, the incorporation of CNC showed an irregular trend in the values of the melting temperatures due to possible variations in the crystalline domains that each concentration of CNC induced on the polymeric matrix. In the case of 0.5% and 5% (*w*/*w*) CNC, the fusion of the starch crystallites was observed around 155 °C; some authors have reported higher values (at 160 °C) [42]. The shift of this thermal transition slightly towards lower temperatures was probably due to the plasticizing effect of the CNCs on the matrix, which means that lower temperatures are needed to melt these types of nanocrystals.

### 3.5. X-ray Diffraction

**Figure 4:**
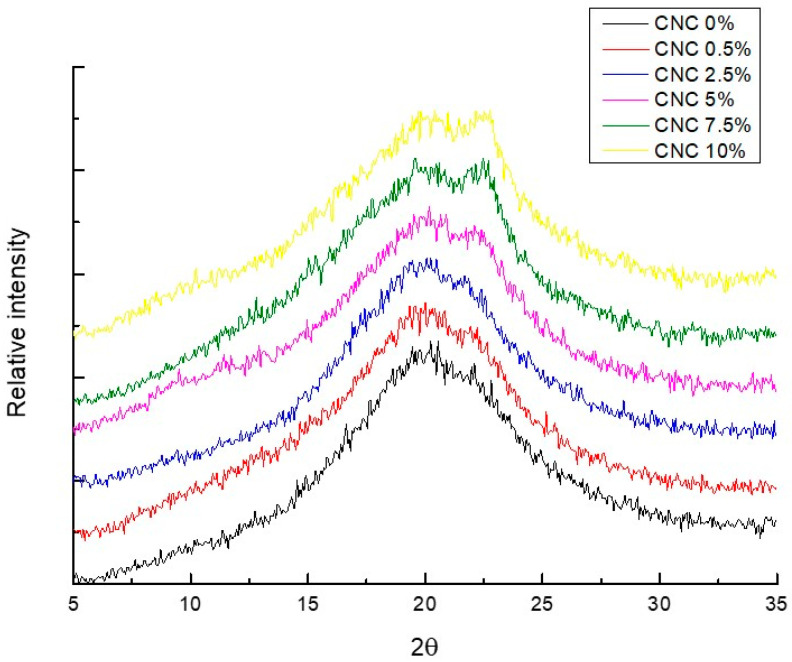
X-ray diffractograms of starch–chitosan films reinforced with CNC (0 to 10% *w*/*w*).

Figure 4 shows the X-ray diffraction patterns of biodegradable films based on starch–chitosan with several proportions of CNC. In the starch–chitosan biodegradable films, the presence of a peak at 22.42° was an indication of the crystalline nature of the CNC nanoparticle [6,20,42], being more visible when the concentration of nanocrystals was 2.5% (*w*/*w*) and higher. These nanocrystals correspond mainly to type I cellulose, which has a high crystallinity index, as observed through a diffraction peak at 20.5° [43], which was visible in the films developed in this study with CNC contents of 5, 7 and 10% (*w*/*w*). In the control films, a behavior corresponding to a more amorphous material was observed, which became more crystalline, that is, more ordered with the incorporation of the CNC. The crystalline nature of nanocellulose helps to generate packaging materials with a more ordered structure; this is reflected in the physicochemical characteristics of the material, i.e., mainly in the thermal, mechanical, and water absorption properties, which are described in Section 3.4, Section 3.6 and Section 3.7.

### 3.6. Water Vapor Adsorption Isotherms

The adsorption isotherm graphically represents the relationship between water activity (or the equilibrium relative humidity of air in the environment) and material moisture content under conditions of equilibrium and constant temperature. It also allows us to know how water binds to the material [13,44]. Various mathematical models provide information on parameters that could effectively monitor a material or food during its storage [30]. The films composed of starch and chitosan presented a water absorption capacity that changed depending on the concentration of CNC.

**Figure 5:**
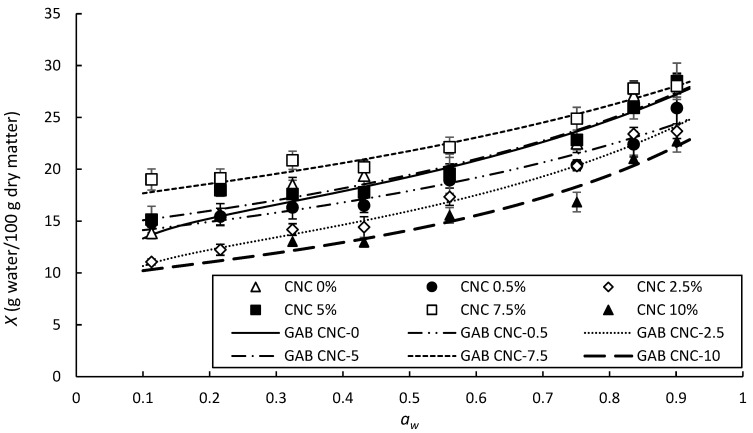
Water adsorption isotherms of starch–chitosan films reinforced with CNC (0, 0.5, 2.5, 5, 7.5, and 10% *w*/*w*) at 25 °C. Experimental data (symbols); fitted to the GAB mathematical model (solid and dotted lines).

By incorporating CNC into biobased starch–chitosan materials, their water absorption capacity decreased, except when 7.5% CNC was added (Figure 5), as illustrated in the relevant X-ray diffraction pattern. The higher the crystallinity of the material, the lower the water absorption capacity, because water molecules first interact with the amorphous part of the material. However, the shape or type of isotherm was not modified with an increase in the content of nanomaterials, with each film maintaining the same behavior or slope in the total range of relative humidity used in the analysis of the water adsorption isotherms. It has been reported that when the proportion of chitosan increases in starch-based formulations, the shape of the isotherm and the moisture content that these materials can absorb increase significantly when subjected to RH conditions higher than 60% or *a_w_* 0.6 [13]. According to Brunauer [45], the isotherms of the films evaluated in this study corresponded to those of type 2, where the adsorbate (water molecules) covers the film (adsorbent) until a monolayer is formed and the adsorption process continues in the form of a multilayer. This is a common profile in physical adsorption processes in which interactions are not very specific. Therefore, to produce this type of behavior, the affinity of the adsorbate for the adsorbent must be higher than the affinity of the adsorbate for itself [45,46]. In this type of isotherm, two identifiable regions (i.e., two different slopes on the curve) can be observed: one around *a_w_* 0.1 to 0.4 and the other around *a_w_* 0.6 to 0.9. This represents typical behavior of a regular food product [46]. All materials showed an increase in the amount of water they could absorb when subjected to high relative humidity, i.e., mainly in regions with *a_w_* from 0.8 to 0.99. This range of *a_w_* is a critical point for the preservation of food products due to the presence of free water, which is no longer directly linked to the material and is more available so that pathogenic and deteriorating bacteria can perform their vital functions and reproduce [46,47]. Foods within this range of *a_w_*, such as eggs, meats, vegetables, and fresh fruits, are therefore potentially more susceptible to harboring pathogenic bacteria.

In this study, a reduction in the content of water that the material could adsorb was observed with an increase of CNC content in the structural matrix, with the sample containing 7.5% CNC being able to absorb even more moisture than the control film. This indicated that in this formulation, the polymeric chains were free, interacting little with each other, making them more available to bond with water molecules. This was reflected in the parameters calculated by the GAB mathematical model, as shown in Table 3.

The value of *Xm* for the sample with 7.5% CNC presented the highest value, with a water content of 17% forming the monolayer of water that interacted directly with the material. In the monolayer, it was assumed that each hydrophilic group on the material had a bound water molecule [46]. This water content, present in the monolayer phase, was much higher than that of the control film, as well as that absorbed by the films containing lower percentages of CNC, although its binding energy was lower, as expressed by the *K* parameter.

### 3.7. Mechanical and Gas Barrier Properties

In addition to their antimicrobial capacity, nanocomposites have been reported to improve the barrier properties against gases (water vapor and oxygen) and the mechanical properties of natural polymer-based packaging materials [16,48]. Table 4 shows the mechanical properties (tensile strength and elongation at break) and water vapor permeability of corn starch–chitosan films reinforced with CNC.

A significant improvement in the mechanical properties of tension and elongation at fracture was observed with the addition of cellulose nanocrystals. Without presenting any trend or pattern of behavior as CNC concentration increased, in the present study, something similar to what was observed in other characterizations of these materials was observed. The addition of CNC at concentrations of 2.5% to 10% significantly increased the strength of the material, with the sample with 7.5% showing the highest value, i.e., 13.61 MPa, while the control (0% CNC) was 3.49 MPa. Regarding the elongation of the material, the film without CNC presented an elongation of 74.67%. When 0.5 and 5.0% of CNC were added to the formulation, the material increased its extension up to 140 and 145%, respectively. However, it was found that when nanocrystals were added in a proportion of 7.5%, the material was significantly affected, reducing its elongation to a value of 5.32%. In cassava starch films obtained by the casting method, it was found that incorporating only 1.5% of CNC (modified with stearic acid) improved the tensile strength of the materials by more than 300% [20]. This result was attributed to the fact that cellulose in micro- and nano-sizes has a high resistance to tension. When nanomaterials are compatible with the polymer matrix, a strong matrix-filler interaction is generated due to Van der Waals forces that can transfer stress, thus improving the resistance of the materials [49]. Nonetheless, the mechanical performance of the starch–chitosan-CNC films still cannot be compared to those of some films of synthetic origin, such as polyethylene [50].

As observed in the SEM microphotographs in Figure 2b–e, it is likely that the nanomaterials agglomerated when added in higher proportions [51]. These agglomerates may have influenced the mechanical properties of the materials. An excess of nanocomposites such as CNC negatively modified the mechanical properties of packaging materials [20], so it would be necessary to determine the optimal concentration according to the results or properties sought for a specific material.

According to the results obtained by Maradini, Oliveira [52], the tensile strength and Young’s modulus of polyester can be improved with the addition of 1% (*w*/*w*) of CNC; however, when the concentration is increased to 2%, the mechanical performance is negatively affected. Although the addition of 4.5% CNC in PLA-gelatin multilayer films significantly reduced the tensile strength and elongation, this nanocomponent was not a viable alternative to reinforce this polymeric matrix [22]. When the nature of the reinforcement material and the polymeric matrix are not the same, this low compatibility can be seen as a weakness in terms of mechanical properties, i.e., the same effect as that generated when the nanomaterials have not been uniformly dispersed in the structural matrix material [53].

Water vapor permeability (WVP) is directly influenced by various material parameters, such as the hydrophilic or hydrophobic nature, the presence and type of additives, as well as the morphology of the resulting materials. Table 4 shows the WVP of the films made in this study based on their NCN content. Films containing 2.5% CNC (*w*/*w*) showed a lower WVP (2.46 × 10^−10^ g·m^−1^s^−1^Pa^−1^) than the control film and those with other nanocrystal concentrations. This behavior can be attributed to the hydrophobic and highly crystalline nature of the cellulose nanocrystals, in contrast to those of starch and chitosan. The addition of nanometric-sized additives can improve the structural matrix of biopolymers, generating a tortuous path and making it difficult for water molecules to pass. Other authors have reported similar results for starch-based biodegradable films reinforced with different nanometric particles [16,54]. However, a large amount of CNC could be considered excess, encouraging these nanomaterials to agglomerate and giving rise to a less homogeneous film, causing gases to flow more freely through the polymer chains and increasing their permeability [26,39]. Azeredo, Mattoso [55] reported that the presence and increase in the concentration of cellulose nanofibers in chitosan-glycerol films caused a significant reduction in WVP. A similar result was reported by Chou, Shi [56], who noted that the WVP of PVOH films decreased with the addition of CNC at 2.5% (*w*/*w*). Meanwhile, for biodegradable films made from a mixture of cassava starch, chitosan and gallic acid, the incorporation of 7.5% (*w*/*w*) cellulose nanofibers generated the material with the best water vapor barrier capacity [39].

## 4. Conclusions

This study made described a method for the production of biobased films from a mixture of corn starch and chitosan, which served as carriers of reinforcing nanocomposites, such as cellulose nanocrystals (CNC). It was observed that the cellulose nanomaterials were arranged differently with the polymeric chains of starch and chitosan, as reflected in their mechanical, rheological, thermal, and gas barrier performance. The small size of the CNCs helped them mix easily with the polymeric matrix; however, this interaction was limited to a certain extent. It was observed that when more nanomaterials were added, they could not disperse uniformly, which negatively affected mechanical performance, as manifest in a decrease in tensile stress. According to the results of the calorimetric analysis, a relationship was observed in the 0.5% and 5% CNC samples that presented greater elongation due to the plasticity effect and structural arrangements that allowed the structure recover under tension. The starch–chitosan films with CNC concentrations of between 0.5 and 5% presented the best functional properties, perhaps due to a more uniform distribution of the nanomaterials. The maximum inhibitory effect of the CNC nanocomposite films was observed with *Listeria monocytogenes* bacteria when 0.5% CNC was added. In comparison, the inhibitory impact on *Staphylococcus* *aureus* was very similar to that observed with the control films, which themselves showed a significant antimicrobial effect.

Among the critical factors in the selection of food packaging materials are the permeability and the speed of transmission of gases (water, light, oxygen, ethylene, aromas) and the mechanical performance that determines if the material will be able to maintain the integrity of the product. The films developed in this study were found to be permeable to water vapor to different degrees. The concentration of nanoparticles that can generate useful materials based on starch and chitosan can be determined according to the data presented herein. This study may be the starting point for the production of new nanocomposite materials; such antimicrobial films are currently being tested to preserve fresh fruits which are of economic importance but which are susceptible to the action of bacteria.

## Figures and Tables

**Table 1:** Antimicrobial effect of cellulose nanocrystals (CNC) incorporated in corn starch–chitosan biodegradable films.

CNC Content (*w*/*w*) in Starch–Chitosan Films	Inhibition Halo Diameter (mm)	
	*Listeria* *monocytogenes*	*Staphylococcus* *aureus*
0% (control)	11.02 ± 0.59 ^b^	11.00 ± 0.71 ^b^
0.5%	12.52 ± 0.01 ^c^	8.79 ± 0.49 ^a^
2.5%	11.35 ± 0.49 ^b^	11.32 ± 0.49 ^b^
5.0%	9.39 ± 0.12 ^a^	(--)
7.5%	(--)	(--)
10.0%	(--)	(--)

Mean ± standard deviation of three replicas. Values with different letters (^a, b, c^) in the same column denote significant differences (Tukey test; *p* < 0.05). (--): No inhibition halo was observed.

**Table 2:** Temperature data (expressed in °C) related to thermal analysis of nanocomposite films incorporated with CNC (0 to 10% *w*/*w*).

Starch–Chitosan Film Sample	TGA		DTG	DSC	
	T_10_	T_30_	T_d_	T_g_	T_m_
CNC 0%	166.9	290.7	326.1	54.7	144.9
CNC 0.5%	167.6	303.1	330.3	--	154.3
CNC 2.5%	170.5	296.4	326.9	63.4	122.5
CNC 5.0%	156.8	295.1	328.8	--	156.9
CNC 7.5%	214.6	304.9	327.8	--	120.3
CNC 10.0%	181.6	298.2	329.8	61.9	127.6

(--) It was not possible to determine.

**Table 3:** GAB mathematical model parameters and regression coefficient, R^2^, calculated for composite starch–chitosan films with CNC.

CNC Content (*w*/*w*) in Starch–Chitosan Films	*Xm*	*C*	*K*	R^2^
0%	14.67	151.59	0.53	0.984
0.5%	13.46	6283.33	0.50	0.993
2.5%	11.57	106.25	0.58	0.997
5.0%	14.32	7692.23	0.53	0.987
7.5%	17.04	2863.44	0.43	0.991
10.0%	9.73	958.54	0.62	0.987

**Table 4:** Physicochemical properties of starch–chitosan biodegradable films reinforced with cellulose nanocrystals (CNC).

CNC Content (*w*/*w*) in Starch–Chitosan Films	Tensile Strength (MPa)	Elongation at Break (%)	WVP × 10^−10^ (g·m^−1^s^−1^Pa^−1^)
0%	3.49 ± 0.32 ^b^	74.67 ± 5.48 ^b^	3.01 ± 0.37 ^a^
0.5%	2.89 ± 0.23 ^a^	140.40 ± 10.62 ^d^	4.84 ± 0.55 ^b^
2.5%	6.16 ± 0.39 ^c^	88.96 ± 8.99 ^c^	2.46 ± 0.21 ^a^
5.0%	6.72 ± 0.41 ^d^	145.13 ± 12.74 ^d^	4.33 ± 0.34 ^b^
7.5%	13.61 ± 0.55 ^e^	5.32 ± 0.53 ^a^	4.35 ± 0.26 ^b^
10.0%	6.00 ± 0.50 ^c^	90.57 ± 5.08 ^c^	4.30 ± 0.27 ^b^

Values with different letters (^a, b, c, d, e^) in the same column denote significant differences (Tukey test; *p* < 0.05). Values are given as mean ± standard deviation (n = 8 for mechanical properties, and n = 4 for WVP).

## Data Availability

The data presented in this study are available on request from the corresponding author.
